# Using consumer perspectives to inform the cultural adaptation of psychological treatments for depression: A mixed methods study from South Asia

**DOI:** 10.1016/j.jad.2014.03.036

**Published:** 2014-07

**Authors:** Neil Krishan Aggarwal, Madhumitha Balaji, Shuba Kumar, Rani Mohanraj, Atif Rahman, Helena Verdeli, Ricardo Araya, M.J.D. Jordans, Neerja Chowdhary, Vikram Patel

**Affiliations:** a Columbia University, New York State Psychiatric Institute, 1051 Riverside Drive, Unit 11, New York, NY 10032, USA; b Parivartan Trust, Plot no. 20, Vijaya Villa Survey, No. 235A, Sanjay Park, Lane no. 1, Lohgaon Airport Road, Pune 411014, India; c Samarth, No. 100, Warren Road, Mylapore, Chennai 600004, India; d University of Liverpool, Institute of Psychology, Health & Society, Child Mental Health Unit, Alder Hey Children׳s NHS Foundation Trust, Mulberry House, Eaton Road, Liverpool L12 2AP, UK; e Department of Counselling and Clinical Psychology, Teachers College, Columbia University, 525W 120th Street, New York City, NY 10027, USA; f Academic Unit of Psychiatry, University of Bristol, Oakfield House, Oakfield Grove, Bristol BS8 2BN, UK; g Health Net TPO, Lizzy Ansinghstraat 163, 1073 RG Amsterdam, The Netherlands; h Sangath Centre, 841/1 Alto-Porvorim, Sangath, Goa 403521, India; i London School of Hygiene and Tropical Medicine, Keppel Street, London WC1E 7H, UK; j Center for Global Mental Health, Institute of Psychiatry, King's College London, Box P029, De Crespigny Park, London SE5 8AF, UK

**Keywords:** Depression, South Asia, Explanatory models, Psychological treatments

## Abstract

**Background:**

Integrating consumer perspectives in developing and adapting psychological treatments (PTs) can enhance their acceptability in diverse cultural contexts.

**Objective:**

To describe the explanatory models (EMs) of depression in South Asia with the goal of informing the content of culturally appropriate PTs for this region.

**Methods:**

Two methods were used: a systematic review of published literature on the EMs of depression in South Asia; and in-depth interviews with persons with depression and family caregivers in two sites in India. Findings from both were analysed independently and then triangulated.

**Results:**

There were 19 studies meeting our inclusion criteria. Interviews were conducted with 27 patients and 10 caregivers. Findings were grouped under four broad categories: illness descriptions, perceived impact, causal beliefs and self-help forms of coping. Depression was characterised predominantly by somatic complaints, stress, low mood, and negative and ruminative thoughts. Patients experienced disturbances in interpersonal relationships occupational functioning, and stigma. Negative life events, particularly relationship difficulties, were perceived as the main cause. Patients mostly engaged in distracting activities, religious practices, and received support from family and friends to cope with the illness.

**Limitations:**

The primary data are entirely from India but the studies from the literature review covering South Asia are consistent with these findings. This study also does not include literature in local languages or explore how consumer perspectives change over time.

**Conclusions:**

EMs can inform cultural adaptations to PTs for depression in South Asia by defining target outcomes, content for psycho-education, and culturally appropriate treatment strategies.

## 1 Introduction

A growing scholarship has emphasised the need to culturally adapt evidence-based interventions to promote their implementation and dissemination across a wide variety of clinical practice settings. Cultural adaptations of interventions are defined as “the systematic modification of an evidence-based treatment or intervention protocol to consider language, culture, and context in such a way that it is compatible with the client׳s cultural patterns, meanings, and values” (Bernal et al., 2009). Such adaptations can extend the generalisability of psychological treatments (PTs) that tend to be developed for young, Caucasian, upper middle class populations in high-income countries (Kirmayer, 2012). A number of studies have outlined the benefits of adapting evidence-based PTs to diverse cultural contexts; for example, a recent meta-analysis showed that culturally-adapted PTs were more effective than standard PTs for a range of patient outcomes (Smith et al., 2011).

The incorporation of patient “explanatory models” has been found to be a key element in the process of cultural adaptation of effective PTs (Chowdhary et al., 2013). Explanatory models (EMs) have been defined as “notions about an episode of sickness and its treatment that are employed by all those engaged in the clinical process” (Engelhardt, 1974; Kleinman, 1980). Such notions can be operationalised by treating interviews with patients, families, and community members as mini-ethnographies that explore views on an illness (Kleinman et al., 1978). Eliciting EMs is especially important in public health programs that may fail if social and cultural differences among providers and patients are not bridged (Hahn and Inhorn, 2009). EMs perform several tasks simultaneously: they explicate the cultural implications of illness experiences for patients and providers, promote empathy and therapeutic alliance, and correct the tendency for providers to overemphasise biological models of illness (Weiss and Somma, 2007). The cultural information obtained from EMs can be coupled with evidence-based PTs to effectively integrate treatments within a comprehensive bio-psychosocial paradigm (Bhui and Bhugra, 2002): for example, this information can be contrasted with principles underlying PTs to uncover theories and practices that require adaptation to improve the acceptability of such PTs.

Depression is the leading cause of morbidity worldwide (Lopez et al., 2006). In South Asia, which houses a fifth of the global population, depression is the most frequently reported mental disorder in epidemiological studies. It is associated with social disadvantage, and highly correlated with impaired infant growth (Patel et al., 1998; Rahman et al., 2004) and suicide (Maselko and Patel, 2008). Meta-analyses have established the effectiveness of PTs for depression (Dobson, 1989; Weisz et al., 2006; Cuijpers et al., 2008), including PTs adapted for use in culturally distinct populations (Chowdhary et al., 2013). The World Health Organisation (2010) has recommended that PTs be used as a first-line treatment for mild depression and in conjunction with medication for moderate to severe forms of depression. Although it has been proposed that PTs be scaled up in low- and middle-income countries for use by lay or community health workers in task-sharing models of service delivery to fill the resource gap of therapists with advanced degrees (Patel et al., 2009; van Ginneken et al., 2013), the content and process of delivery of PTs need to be adapted to the local context to enhance their feasibility and acceptability (Chowdhary et al., 2013; Rahman, 2007). While certain PTs such as cognitive behavioural therapy (CBT) have been found to improve symptoms when culturally adapted for ethnic minority patients in the United States (Otto and Hinton, 2006; Hinton et al., 2011, 2012), the process of immigration involving pre- and post-migration stressors and supports may define EMs of American minority patients in ways that are unlikely to affect populations that have never migrated (Bemak et al., 2003). Therefore, the aims of this paper are to describe the EMs of depression in South Asia, from the perspectives of patients, families and community members, integrating data obtained from a systematic review and qualitative interviews, with the goal of informing how evidence-based treatments can be adapted to non-Western cultural contexts. Despite regional, linguistic, and religious diversity, South Asian historians and anthropologists have treated the region as a unit given shared understandings of cultural identity and everyday practice (Bose and Jalal, 1998). For example, governments of many South Asian countries offer training in medical practices to one another, provide humanitarian assistance during natural disasters, and house refugee populations from neighbouring countries (Aggarwal and Kohrt, 2013). Therefore, we explore EMs of depression throughout South Asia, noting similarities and differences where present.

## 2 Methods

This study was conducted through two methods carried out concurrently. The first method was a systematic review of the literature on depression from South Asia. The second was in-depth interviews (IDIs) with patients suffering from depression and family caregivers. Findings from both methods were triangulated to address our research questions. We used this mixed methods approach to strengthen the richness of our findings and enhance data validity (Thomas et al., 2004; Owen et al., 2010; Rowe et al., 2012; Lawrence and Kinn, 2012). While the systematic review provided a summary of the literature to date, the IDIs provided a more detailed understanding of individual phenomena and filled conceptual gaps in the literature. We explain the process of each method below.

### 2.1 Systematic review

This systematic review was undertaken in accordance with a review protocol. The relevant literature on PTs for depression was identified in four ways. First, we searched the English language electronic databases PubMed Central, PsycInfo, PsycExtra, and IndMed (an index of Indian medical journals), pairing the terms “depression” and “depressive,” with “coping,” “help seeking,” “self-help,” “explanatory model” or “illness narrative.” We decided to include the terms “coping,” “help seeking,” and “self-help” to examine what consumers actually tend to do since EMs focus on the names, causes, fears, and problems, as attributed by consumers to an illness (Kleinman, 1980). This search was conducted by two researchers [NKA and NC] independently in December 2010 and March 2011 to check the reliability of our searching strategy.

Second, we searched the bibliographies of selected articles for additional literature. Third, we approached key informants to recommend relevant articles and the names of additional informants. Fourth, we visited leading Indian institutions to search the table of contents of journals not indexed in the databases (such as the Indian Journal of Social Work) and to access the “grey” literature such as books, project reports, manuals, and dissertations. All search results were downloaded into bibliographic software and screened by two researchers [NKA and NC] with at least 10% receiving a quality check using predefined inclusion and exclusion criteria from the search protocol. The inclusion criteria were as follows: all papers that reported original data from South Asia, defined as India, Pakistan, Bangladesh, Sri Lanka, Bhutan, Nepal, Maldives, and Afghanistan; literature published in English from January 1990 to March 2011; and studies that reported EMs for depression in adults, defined as subjects older than 17 years old. The exclusion criteria were as follows: studies that did not present original research; enrolled subjects with comorbid disorders such as adjustment or substance use disorders; and did not report experiences of depression.

Articles of interest were identified by reading titles, followed by retrieving and scanning abstracts. All relevant citations were retrieved in full when possible. Authors were contacted when full texts were not available online. Two attempts were made to contact each author through email, telephone or post. Two researchers independently assessed each retrieved paper [NKA and NC]. An independent researcher [VP] blind to the review examined all papers to validate inclusion criteria and to rescreen 10% of the rejected papers by reviewing titles and abstracts.

**Fig. 1:**
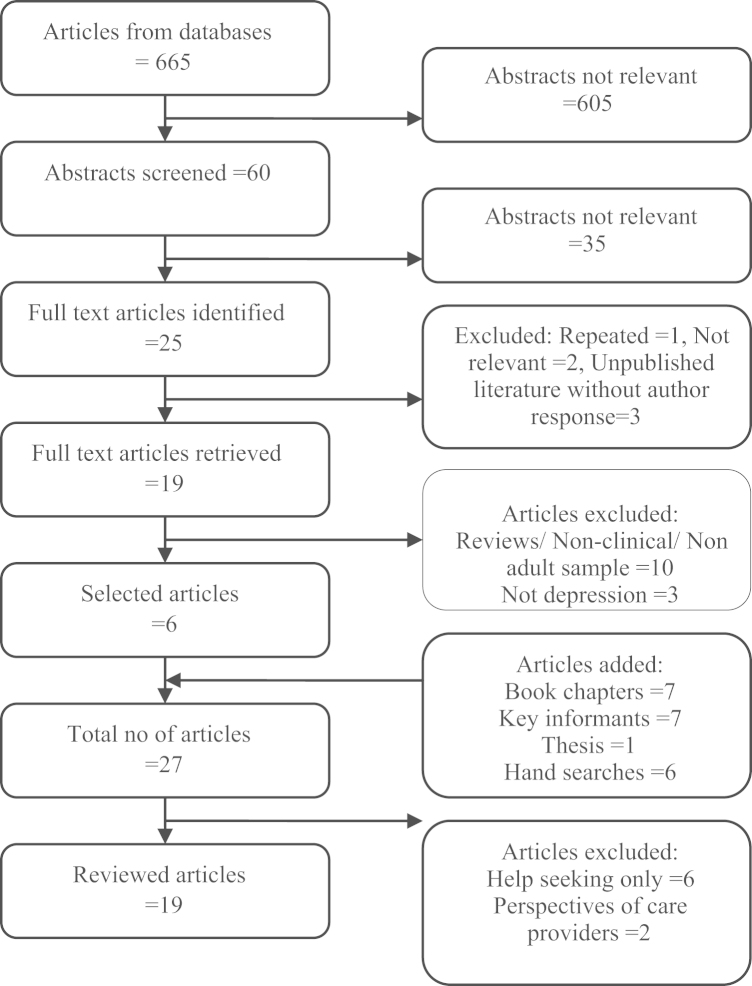
PRISMA flowchart on included studies of explanatory models for depression in South Asia.

Data from included papers were abstracted into Microsoft Excel spreadsheets. A structure was developed to analyze each study based on variables of interest such as study design, aim, study population setting, sample size, and outcomes. Three researchers reviewed a random subset of papers and reached consensus on a coding template; this was based on Kleinman׳s EM framework to include additional outcomes, when reported, that were related to (1) illness descriptions, (2) the perceived impact of the illness, (3) causal beliefs about the illness, and (4) self-help coping strategies. Two researchers (NKA and NC) then coded all of the articles independently to minimise bias by copying text verbatim into the spreadsheet and then coding this text according to the EM framework. Both researchers are psychiatrists with extensive experience in social science methodologies. They undertook peer-debriefing activities to detect coding differences and a third researcher (VP) resolved the differences. The third researcher also has extensive experience in qualitative coding, and supervising medical trainees and graduate students in qualitative data analyses. Within the four EM codes, the generation of sub-codes was inductive based on raw data. This review conforms to the Preferred Reporting Items for Systematic Reviews and Meta-Analyses (PRISMA) (Moher et al., 2009). A PRISMA flowchart of the numbers of studies at each stage is presented in Fig. 1.

### 2.2 In-depth interviews

We conducted IDIs (Holloway and Wheeler, 2009) with persons with depression and their caregivers in sites in two Indian states (Goa and Maharashtra). Adults with a current (“ill”) or past (“recovered”) history of depression who met the ICD-10 diagnostic criteria or scored above a validated cut-off score on the General Health Questionnaire (Patel et al., 2008) were eligible to participate. A caregiver was any family member of a person with depression who lived with and cared for him/her during the illness. To capture potential variations in EMs for depression, we used maximum variation sampling (Miles and Huberman, 1994). We did this by selecting subjects who accessed treatments from a variety of health care providers, including mental health specialists (such as psychiatrists), non-specialists in the biomedical health system (such as general practitioners), and providers outside of the biomedical health system (such as religious healers or yoga and meditation trainers).

Interview guides were developed, one each for persons with depression and caregivers. These were developed by a research coordinator (MB) based on research questions with guidance from qualitative experts (SK and RM). MB is a clinical psychologist and mental health researcher who has over five years of training in qualitative research. SK and RM have worked on several studies on mental health, HIV, women׳s health and have had over 10 years of experience in qualitative research. Questions in the guide were open-ended, with the interview flow determined by information from participants. A sample of the questions is given below.

• Can you tell me about your problem?
• What do you call this problem?
• What according to you has caused the problem?
• How has the problem affected your life? What has changed in your life after the problem started?
• What do you do to manage/cope with this problem? Can you describe what all you do to make yourself feel better? How has this helped you?

These guides were translated from English into the local languages of Konkani and Marathi. Konkani and Marathi are closely related languages and spoken by geographically contiguous populations; some linguists have even contended that the two languages can be seen on a spectrum given their shared grammar and vocabulary (Masica, 1991). Therefore, while there may be cultural differences, we would not expect these to be relevant to the linguistic expressions of the experience of depression.

Participants were interviewed in person either at home or in treatment facilities by trained researchers. All interviews were conducted in English, Konkani or Marathi by research staff fluent in these languages. They were audio taped, transcribed verbatim, and translated into English. To ensure the quality of interviewing, MB examined the first 10 IDIs for richness of information, open-ended questioning, and probing style, giving researchers detailed written feedback. Three randomly selected transcripts were then sent to the qualitative experts (SK and RM) for review and comment. Thus, there were two levels of supervision to maximise interview quality.

Data from the IDIs were entered into and coded in Nvivo 8 (QSR International Pvt. Ltd., Australia). We used thematic analysis to analyse the information (Braun and Clarke, 2006). Analysis was at first deductive, consisting of predetermined codes based on original concepts from Kleinman׳s EM framework (Kleinman et al., 1978; Kleinman, 1980), for example “Causal beliefs.” This formed the initial coding template from which four IDIs were coded. The template was then revised through investigator consensus and the new coding template was applied to the remaining interviews. Subsequent analyses were inductive and focused on the generation of new codes from raw data. At this stage, codes were iteratively compared and contrasted: similar codes were collapsed into inclusive categories; additional codes were integrated into the coding template; and clusters of related codes were organised under other codes, forming hierarchies. For example, narratives describing a patient׳s visits to temples, churches or other places having religious deities as a way of coping with their illness were coded as “visiting places of religious worship”. These codes were then combined with other codes judged to be similar in meaning, such as “prayer” or “reading religious texts”, and collapsed to form a category, ׳Religious/spiritual practices׳ This, along with other categories of coping, such as “Solving Problems” was organised under “Self-Help Strategies”.

The lists of coded hierarchies from each method were extracted and then compared and contrasted. Codes that were similar were collapsed. Additional codes (from either method) were retained. Themes were derived by retrieving pieces of data pertaining to codes and by examining their meaning in relation to the research questions.

The Institutional Review Boards at the London School of Hygiene and Tropical Medicine and Sangath approved the study.

## 3 Results

Nineteen studies were included in the review, 14 of which were from India, 4 from Pakistan, and 1 from Nepal. Fig. 1 presents the PRISMA flowchart on the sources of these studies and Table 1 lists all study characteristics by study design, aim, study setting, sample characteristics, sample size, and major findings. Investigators used a wide range of study methods such as qualitative interviews (*n*=13), case-control comparisons (*n*=4), and surveys (*n*=2). Four studies enrolled community samples whereas fifteen studies enrolled people with depression identified through diagnostic interviews or symptom questionnaires. Sample sizes ranged from 7 to 901. Twelve studies provided information on illness descriptions, 8 on perceived impact, 13 on causal beliefs and 9 on coping strategies.

IDIs: There were 27 people with depression and 10 caregivers (Table 2). Eight caregivers were relatives of interviewed patients. The mean ages of persons with depression and their caregivers were 45 and 43 years respectively. About two-thirds of the sample were female (*n*=17). Nearly 60% had completed high school and 41% were employed. Two-thirds (*n*=18) had fully recovered from depression. Seven had completed high school and four were employed.

Below, we present results by coding theme, incorporating findings from both methods. We coded four main themes as follows: (1) illness descriptions, (2) perceived impact of the illness, (3) causal beliefs, and (4) self-help coping strategies.

### 3.1 Illness descriptions

#### 3.1.1 Phenomenology of the illness

People with depression frequently described their illnesses as physical conditions to health providers (Rahman, 2007; Weiss et al., 1995; Mirza et al., 2006; Shankar et al., 2006; Pereira et al., 2007; Prameela et al., 2007; Naeem et al., 2004; Savarimuthu et al., 2010). In one study, 21% of 80 depressed outpatients reported suffering from aches and pains and 58.8% reported that somatic symptoms were more troubling than depression (Weiss et al., 1995). In another study of 35 married women with depression (Pereira et al., 2007), the most common illness descriptions included musculoskeletal aches and pains, autonomic disturbances, fatigue, headaches, palpitations, and/or weakness. Many patients believed that their symptoms reflected a “physical disease” (Rahman, 2007; Pereira et al., 2007). Our IDIs showed similar findings. For example, a 48-year old man with depression described his illness in somatic terms: “*My head used to become heavy and I used to feel like there were ants crawling (in my head)*… *I used to feel numbness*.” Family caregivers also reported physical health complaints. A 37-year old daughter-in-law of a woman with depression described her as having “*difficulty in swallowing*”, and “*problems of breathlessnes*s,” and a 38-year old wife described her husband׳s symptoms as something happening to the “*nerve behind his head*.”

Mood symptoms were reported in two studies as “sadness” and “psychological distress” (Mirza et al., 2006; Chetna, 2005) and ruminative thinking was observed in two other studies (Pereira et al., 2007; Sharma, 1998). Depressed persons frequently experienced self-reproach, and helplessness, fearing that their future could not improve (Rahman, 2007; Chetna, 2005; Sharma, 1998; Verma, 1989; Chandrashekhar et al., 2007). Low self-confidence was reported in one study (Rahman, 2007) and suicidal attempts were reported in another (Shankar et al., 2006).

Such symptoms were also observed in our IDIs. For example, a 65-year old mother of a woman with depression noted that her daughter used to feel “*crestfallen*, *like everything was over*” and “*there was nothing to live for*.” People with depression and their caregivers reported that patients were irritable, refused to eat, had low self-confidence, could not concentrate or sleep, and experienced negative thoughts. A 48-year old man with depression reported: “*Everything (is) negative. What I am thinking will not work. Whatever I do won׳t help. There is a lot of negativity in me.*” Ruminative thinking and anxiety, often involving past trauma or worries about the future, were prominent. A 26-year old woman with depression said: “*I haven׳t told anybody this until now, but I suffered sexual harassment, and it is still there in my mind! I was unable to sleep. It has no relevance to my life now, but I used think about the same thing all night.*” A 28-year old man with depression said: “*Thoughts keep on revolving in my mind. Even if I sit watching TV, thoughts come in my head*…. *like* ‘*Will I be able to take care of my house?*’”

#### 3.1.2 The labelling of the experience

Many people with depression used somatic terms to label their illness (Weiss et al., 1995; Mirza et al., 2006; Shankar et al., 2006; Pereira et al., 2007; Prameela et al., 2007; Naeem et al., 2004; Savarimuthu et al., 2010). In one study, 18.8% of 80 Indian outpatients called their problem an “illness of the body” and 10% labelled it a “nerve problem” (Weiss et al., 1995). In a study from Pakistan, all 11 people with depression attending a primary care clinic believed that their body was affected by depression (Mirza et al., 2006). In two studies, depression was specifically labelled as “stress”, “tension,” or “pressure” (Rahman, 2007; Naeem et al., 2004). Only in a minority of studies did outpatients report depression as a mental illness. In one study of 25 outpatients in India, 11 labelled their problems as an “illness of the mind” and 3 called it “illness of sadness” or “illness of the mind and body” (Weiss et al., 1995). Such perceptions were also held by community members (Kermode et al., 2007, 2009a). In one study, researchers presented a depression vignette to 240 households in India and found that 55.5% identified symptoms as “depression,” 47.1% “a mental illness,” 33.3% “a mind/brain problem,” and 28.3% “a psychological/emotional problem” (Kermode et al., 2009a, 2009b).

The names by which IDI participants referred to the illness ranged from terms such as *“worry”*, *“tension”*, *“pressure”*, *“nerve in the brain”*, *“stress”*, *“emotional blockage,”* and *“mental problem”* to “*depression”* and *“anxiety.”* Some people did not know that they were suffering from *“depression”* until much later. A 37-year old man with depression stated: “*I had depression, in other words, mental stress. Due to work and problems at home, I was unable to sleep. I didn׳t feel hungry, I felt irritated... I asked myself what I could do, but I was unable to understand exactly what treatment I should take. I am in the habit of reading, and I understood from a magazine that many of our illnesses can be psychological. So I mustered my courage to meet with a psychiatrist.*”

### 3.2 Perceived impact of the illness

#### 3.2.1 Interpersonal problems

Three studies reported that depression affected the person׳s ability to fulfill roles within the family (for example, caring for children) and caused interpersonal difficulties (Rahman, 2007; Pereira et al., 2007; Prameela et al., 2007). For example, women with post-partum depression in Pakistan frequently reported isolation from members of the extended family (Rahman, 2007). Our IDIs also illustrated this finding. A 56-year old man with depression explained: “*I started getting very angry with my wife and children. At that time, we were in a joint family (with) my two other brothers, but now we have separated because of my angry and irritable nature.*” A 35-year old man with depression compared himself to a “flower pot” with family members “*only look(ing) at it, not ask(ing) it anything*” since his family avoided engaging him to prevent quarrels.

#### 3.2.2 Social and occupational impairments

Three studies reported that depression affected the performance of routine household and daily activities as well as social and occupational functioning (Mirza et al., 2006; Pereira et al., 2007; Prameela et al., 2007). For example, a study of 11 depressed people in Pakistan found that 5 encountered difficulties with returning to work (Mirza et al., 2006). IDI participants also spoke of this, with one 51-year old depressed woman disclosing: “*I feel sad and do not feel like going anywhere. When I go outside, I feel tired and I am not interested in staying there for a long time.*”

#### 3.2.3 Stigma and discrimination

Two studies reported family and community stigma against people suffering from depression (Shankar et al., 2006; K ermode et al., 2009b). In one of them, stigma was experienced against the self as 30.6% of 72 patients believed that no treatment could help them (Shankar et al., 2006). In the IDIs, people with depression reported that close family and friends often perceived depression as not needing any medical intervention. A 41-year old woman explained that people often did not see depression as a “problem”: “*(If) somebody is depressed, they think that they (persons with depression) have behaved badly and they have control over it. My own mother-in-law would tell me:* ‘*You have to control yourself! How can you be like this?! It is completely in your hands.*’” The fear of how family and friends would react led people to conceal their illness. A 37-year old man with depression said: “*I have told people at home that I am taking a treatment for headaches. It is meaningful to tell someone only if he is going to believe you about a mental problem.*” While the systematic review elicited experiences of stigma at a given point in time, the IDIs provided additional data on the long-term impact of stigma.

#### 3.2.4 Other impacts

The IDIs also demonstrated long-term impacts of depression that were not mentioned in published studies. For example, a 26-year old woman with depression complained of the “*large amount of money spent on doctors and clinics.*” A 56-year old man explained how depression affected his physical health: “*My health complaints have increased. I have started having problems like sinus [aches], asthma and acidity. These complaints started after my stress.*”

### 3.3 Causal beliefs

#### 3.3.1 Negative life events and difficulties

Six studies reported that people with depression often traced the origins of their illness to negative life events (Weiss et al., 1995; Mirza et al., 2006; Shankar et al., 2006; Pereira et al., 2007; Prameela et al., 2007; Suhail, 2005). Frequently reported causes in the systematic review included interpersonal problems such as conflicts with spouses or in-laws, the lack of social support from family during difficult times, or concern about behaviours of children and family members (Weiss et al., 1995; Mirza et al., 2006; Shankar et al., 2006; Pereira et al., 2007; Prameela et al., 2007; Sharma, 1998). For example, some people attributed their depression to difficulties in finding spouses for daughters or providing suitable care to their children (Prameela et al., 2007; Naeem et al., 2004), while others attributed it to “love failure” (Sharma, 1998) or the death of a family member (Pereira et al., 2007). Interpersonal causes were also reported in the IDIs. The 41-year old female with depression mentioned above spoke of longstanding problems with her parents: “*My mother is a patient with some kind of mental health problem. It was affecting me, especially after my marriage and after my child was born. I had to go back home very often and take care of them (parents). That was creating lot of friction between me, my husband, and daughter. I would get very depressed. There was pressure to do something for the family and I was not able to do that.*”

Financial difficulties also commonly triggered depression. In one study, 24 out of 35 depressed women believed that worries about household finances caused depression (Pereira et al., 2007). In another study, women who had recovered from depression reported concerns about not having enough economically productive family members as a major stressor (Naeem et al., 2004). Financial worries were also reported in the IDIs. One 54-year old man with depression explained: “*My income has decreased. But I can׳t cut down my expenses. My son is still studying. I have to pay his tuition and school fees. It is becoming very difficult for me to keep my head above the water.*”

Work and household stressors were considered to cause depression. In one study, depressed women attributed depression to excessive housework (Pereira et al., 2007). In another study, women were concerned that household stressors could accumulate and lead to depression (Naeem et al., 2004). Interviews with depressed people also exhibited similar concerns. A depressed, 37-year old man said: “*Life is full of hustle and bustle. There is no profession without struggle and mental stress. If the children get poor (exam) marks, we have tension. If the electricity goes, we have tension. We burden ourselves with too many expectations.*” Multiple stressful circumstances, often occurring simultaneously, were also seen as causes. A 39-year old woman echoed such concerns: “*My father was very important to me. He suddenly fell ill and was diagnosed with dementia. My mother couldn׳t handle it, she went into depression. Then she recovered but developed (high blood) pressure, diabetes, psoriasis, and now Parkinson׳s. My job also was proving difficult. I began to feel more and more and hopeless about my circumstances and realized that I was becoming depressed*.”

#### 3.3.2 Religious and supernatural causes

Four studies reported religious and spiritual causes of depression such as negative karma, punishment by God, evil spirits, and black magic (Rahman, 2007; Shankar et al., 2006; Savarimuthu et al., 2010; Suhail, 2005). The most frequent of these was karma. In one study, women with post-partum depression attributed their illness to fate or past actions (Rahman, 2007). However in the IDIs, only one 48-year old man with depression believed that his illness was due to an evil spell: “*I won the lottery. I got a new vehicle. Then I fell ill after that… Someone had done black magic – that׳s why this happened.*”

#### 3.3.3 Physical/medical causes

Two studies reported physical causes of depression. In one study, 22 out of 35 depressed women attributed their illness to reproductive and gynaecological problems such as infertility or sterilisation, and five women to other physical problems such as surgical operations (Pereira et al., 2007). In another study, over 20% of community respondents believed that a virus or an allergy had caused depression (Suhail, 2005). Similar perceptions were evident in the IDIs.

#### 3.3.4 Other causal beliefs

One study reported that 65% of community respondents attributed depression to childhood problems (Suhail, 2005). In two surveys, over 60% believed that depression was caused by “moral weakness” or “personal weakness” (Kermode et al., 2009a, 2009b; Suhail, 2005). In the IDIs, loneliness was perceived as a reason for the illness. A 34-year old woman with depression described her experience: “*At my mother׳s house, we were a joint family. There are lots of guests who come there. Here (at her in-laws house), I have absolutely nobody. Due to stress and loneliness, I became really ill.*” Failed aspirations were also perceived to cause depression. A 28-year old woman explained: “*I tend to curse myself... I am still unmarried and all of my friends have gotten married and have their own kids. So I had this (inferiority) complex. There were lots of questions in my mind as to why I was still unmarried when everybody was getting married. Why am I stuck despite of having so many career options?*”

### 3.4 Self-help forms of coping

#### 3.4.1 Distracting activities

Four studies reported that people with depression engaged in activities they enjoyed to distract themselves, such as watching television, working, listening to music, and reading (Pereira et al., 2007; Chandrashekhar et al., 2007; Job, 2004; Kaikhavani and Kumar, 2005). These findings were confirmed in our IDIs, with people using housework, interests, hobbies, and time with family and friends to keep busy. A 38-year old woman with depression said: “*I started making decorative candles, chocolates, cakes, other things. I used to feel happy doing that to keep myself busy. I was doing something which I like. Since I had to use my mind and concentrate, my mind started becoming steadier. I was not thinking about uncertainty. I was just making candles, being there in the moment.*” A 45-year old wife spoke of her husband: “*Most of the time he reads books in the evening. He thinks less about his tension because of his reading. It helps him engage himself in something instead of just sitting and thinking about a problem.*”

#### 3.4.2 Religious/spiritual practices

Five studies reported that people commonly prayed to cope with their problems (Pereira et al., 2007; Prameela et al., 2007; Naeem et al., 2004; Verma, 1989; Job, 2004). Nearly all persons with depression in our IDIs prayed to God, conducted religious rituals, visited places of religious worship, or attended meetings held by spiritual and religious leaders. A 26-year old woman with depression said: “*Whenever I feel tense, I do meditation in front of God. Uttering his name can at least give you basic trust that somebody is there behind you to support you*.” Others described listening to devotional songs or reading from religious texts such as the Bhagvad Gita or the Bible. A 45-year old man with depression elaborated: “*The thoughts and philosophical knowledge in these books helped me during my period of stress. I started making decisions in a better way. It also helped me in developing business relations and talking to people in business.*”

#### 3.4.3 Support from family and friends

Five studies reported that people with depression sought support from family and friends (Pereira et al., 2007; Naeem et al., 2004; Sharma, 1998; Verma, 1989; Kaikhavani and Kumar, 2005). Social support often took the form of seeking “reassurance” from others in their social network (Naeem et al., 2004; Verma, 1989) or joining self-help groups (Pereira et al., 2007). This form of coping was observed in many persons in the IDIs; people with depression spoke about their problems to and spent time with others. One 35-year old woman with depression reported: “*I go to my friend׳s place, I sit for some time, pass my time. I go take her for a stroll, we come back home, we speak to each other like* ‘*this thing happened and that thing happened,*’ *I tell her* ‘*today I am not feeling well*’”. Family members and friends were often described as “*supportive*”, “*good-natured*” and “*understanding*.” A 56-year old man with depression said: “*My wife always motivates me to get treatment and counsels me that I will feel better after treatment. She also tells me, ‘We have not done anything wrong thing in our life so God will always do good things for us. We will try to get over it by being patient*.’” A 59-year old father reported what he did to help his son with depression: “*I always keep motivating him to do work by saying, ‘You are intelligent and you׳ll certainly be able to do it!*’ *We (wife and I) do everything according to his liking so that he doesn׳t get angry.*”

One study reported that 92% of women with depression kept their feelings to themselves (Verma, 1989). Some persons in the IDIs also attempted to handle problems by themselves. A 45-year old depressed man reported that he did not want help from his family because “*they were not aware about (my) mental health and could not understand it*.”

#### 3.4.4 Positive thoughts and acceptance of life׳s adversities

Three studies reported that people with depression coped by accepting the difficulty of their circumstances or reframed circumstances positively (Pereira et al., 2007; Naeem et al., 2004; Kaikhavani and Kumar, 2005). In a study of 75 women with depression in India, 97.3% accepted that nothing could be changed and over 90% believed that their circumstances would improve with time (Verma, 1989). This was also seen in the IDIs; persons with depression sought comfort in that others had similar problems or were worse off, that one had to be thankful for what one had, and that there were more important aspects to life. A 45-year old man with depression elaborated: “*Roses have thorns but we never wish for roses without thorns. This (illness) is like that.*” Another 54-year old man with depression stated: “*If there is financial stress, I don׳t worry. Being scared won׳t help. I think of many people whose financial positions are worse than ours and there are many people whose financial positions are better than ours. One must be content with what one has.*”

#### 3.4.5 Solving problems

Five studies reported that people with depression often tried to solve problems as a form of self-coping (Sharma, 1998; Verma, 1989; Chandrashekhar et al., 2007; Kaikhavani and Kumar, 2005; Satija et al., 1998). In a study of 75 women with depression, 94.7% tried to analyse their problem and solve them incrementally (Verma, 1989). This finding was confirmed in the IDIs. A 54-year old man with depression stated: “*If the expenses of children increase, we should reduce our (other) expenses to keep expenses within limit.*”

#### 3.4.6 Adopting healthier lifestyles

This finding was reported only in the IDIs. People with depression engaged in healthy behaviours such as eating nutritiously, exercising regularly, and doing yoga or meditation. A 37-year old man with depression said: “*The benefit of exercise is that we don׳t fall sick. Our mentality strengthens and we are ready to face any disaster.*” Persons with depression practiced yoga at home, especially breathing exercises such as Pranayama and Sudharshan Kriya. Such practices prevented boredom and increased positive thoughts. A 43-year old woman with depression added: “*If I rest without doing this (Sudharshan) Kriya, then I start to get angry, feel bored, sit around without doing anything and without having food. But when you do this Kriya, you see so much improvement in yourself on that same day that you can face any kind of difficulty smilingly. You don׳t feel tired with any amount of work you do.*”

#### 3.4.7 Relaxation

This strategy was also reported only in the IDIs. Persons with depression detailed various methods to relax and relieve distress such as doing yoga and meditation (described above), listening to music, reading books, and having a cold bath. Following these activities**,** people reported less “*tension*,” insomnia and anxiety. A 65-year old mother of a woman with depression said: “*Sometimes, she puts on music in the morning and it plays for the whole day. There is a calming effect. When you are angry and put on the music, when there is a nice song, you start humming and forget (problems), you do not have your temper.*”

#### 3.4.8 Self-education

A third strategy reported only in the IDIs related to self-education or bibliotherapy. Some respondents read newspaper articles and books to gather information on the problem and learn how to better cope with depression. A 41-year old woman with depression explained the techniques that she learned to better control her emotions: “*He (the author) has something called the ‘recovery method*’ – *you spot your emotion when it starts going out of control. When you spot it and keep getting into the habit of spotting (it), you will be able to control it.*” People reported not only understanding their illness better, but also feeling more “*fresh*,” “*active*,” and experiencing greater “*energy*”. Some respondents read studies on how others coped with depression for inspiration. Another 41-year old woman with depression extolled the virtues of wise authors: “*When I read the books of good authors, their good thoughts enter my mind. For example, I read the books of Mr. Pravin Davane (Indian writer of poetry, short stories and essays)* . *I learned how to remain down-to-earth from his books.*”

## 4 Discussion

This paper reports experiences of depression in South Asia through a systematic literature review and qualitative interviews with the goal of informing cultural adaptations of PTs. Our results showed that people with depression present with a variety of somatic or interpersonal complaints. These experiences lead to impaired functioning with families and friends, problems with finances and health, stigma, and impaired occupational functioning. The most common causal models related to negative life events, especially with interpersonal relationships. A wide range of self-help coping strategies was reported, the most common being religious/spiritual practices, distracting activities, and seeking support from family and friends.

The findings were generally consistent between the review and the IDIs, but the IDIs reported a wider range of non-somatic illness phenomena and coping mechanisms. These findings have important implications for PTs in South Asia. First, disturbances in multiple domains of functioning indicate the need for PTs to target a variety of outcomes, including low mood, negative thoughts, impaired interpersonal and occupational functioning, and stigma. A combination of strategies may be used. For example, patients who report feelings of resignation and hopelessness may benefit from cognitive strategies while patients who report relationship problems may benefit from interpersonal strategies. Second, the dominance of somatic and stress experiences and idioms indicates the need for service providers to emphasise mind–body connections that facilitate understandings of the relationship between psychological and somatic experiences. Service providers should adopt terms such as “tension” or “stress” to increase patient satisfaction, avoiding formal psychiatric diagnostic labels that may be stigmatising. Thirdly, given the role played by one׳s family in both triggering the illness, as well as helping patients cope with it, PTs should provide psycho-education to families and also involve them in supporting the patients through the PT. Finally, self-help coping strategies which may be contextually appropriate such as religious activities, yoga, and breathing exercises can be incorporated as specific techniques within the PT. Table 3 presents a more detailed description of how illness experiences of consumers can be used to culturally adapt PTs with respect to their goals, strategies, content and delivery.

Our findings of somatic presentations, interpersonal causal models, and self-help strategies are comparable with Asian populations elsewhere and with EMs reported from other low and middle income countries. A study of EMs from depressed women in Iran found that depression was perceived as triggered by negative life events and that coping included seeking help from family, religious practices, and engaging in leisure activities (Dejman et al., 2008). Similarly, a review of EMs of mental illnesses in sub-Saharan Africa found that depressed persons often described illnesses through non-specific somatic complaints (Patel, 1995). Our findings are also comparable with studies on South Asian minority populations in Western countries; for example, South Asians in Canada attributed depression to domestic abuse, marital problems, and family conflicts (Ekanayake et al., 2012). A study in the United Kingdom comparing EMs of Bangladeshi immigrants with British-born natives found that the former attributed illnesses to interpersonal problems whereas the latter deemed their problems medical (Lawrence et al., 2006). Similarly, South Asian immigrants in New York attributed depression to “situational stress” or “life problems”, whereas Caucasian Americans reported biological causes such as hormonal imbalances and neurological problems (Karasz, 2005).

Our study has several limitations. First, although our literature review included journal articles, regional texts, and other grey literature, we did not include articles in local languages. However, it is unlikely that we have missed many articles since peer-reviewed publications from South Asia are typically in English. Second, most studies in the literature review and all respondents of our interviews were from India. Nonetheless, findings from other South Asian countries do not demonstrate inconsistencies. Third, our interviews were conducted with people who accessed treatment and may not represent the wider South Asian community. Finally, newer research suggests that EMs may change throughout the course of illness and may be influenced by treatment, family, friends, and the media (Groleau et al., 2006). As far as we know, no methodology has explored how changing EMs can inform PTs. Future research in South Asia can determine whether EMs, and consumer illness experiences in general, change with, and influence the outcomes of PTs.

This paper is the first to synthesise the literature on EMs and self-help coping with respect to depression in South Asia, and complements this with primary qualitative data. The study demonstrates a number of characteristics which are relevant in the process of adaptation of PT in this region. We recommend an approach to developing PTs which incorporates the views of patients and family members rather than make a priori assumptions about the applicability of “off the shelf” treatments. Such a methodology is consistent with approaches which are grounded within established research traditions in cultural psychiatry and global mental health. The process of adapting evidence-based treatments to cultural contexts in South Asia and the extent to which such adaptations improve patient outcomes present important areas of future research.

## Role of funding source

This research was entirely supported by a Wellcome Trust Senior Research Fellowship to Vikram Patel. The Wellcome Trust (Grant no. 091834/Z/10/Z) does not have any other role in the study.

## Conflict of interest

None of the authors declare any financial or non-financial competing interests.

## Figures and Tables

**Table 1:** PRISMA summary of studies.

**First author, year, study design**	**Aim**	**Study setting by country and sample characteristics**	**Sample size**	**Major findings**
Rahman (2007)	To develop and deliver an intervention to women with mild to moderate depression in the peri-natal period	Two rural sub-districts of Rawalpindi in Pakistan	30 Post-natally depressed mothers	Development of the Thinking Healthy Programme, 16 sessions with each about 45 min, all culturally tailored. Out of 164 women who received the intervention, 156 found it to be useful or very useful. Out of 42 lady health workers trained in the intervention, 37 did not find it to be a burden in their work and 42 understood the concepts in the training.
		Characteristics	24 Lady health workers	
				Illness description
				Depressed women somatised symptoms.
				Perceived impact
				In relationship with significant others, common themes revolved around isolation and ostracism by the family and not living up to expectations.
		Average age 27.5 years	6 Primary care staff	
		All were married		
		44% were uneducated		
		Only 4% were employed		
Qualitative interviews				
Weiss et al. (1995)	To examine the cultural validity of diagnostic categories in South Indian patients	An outpatient clinic of the National Institute of Mental Health and Neuro Sciences (NIMHANS) in India	80 Psychiatric patients presenting for the first time with a diagnosis of depression as diagnosed by staffing physicians	DSM-III-R criteria privilege depressive over somatoform patterns of distress.
				Illness descriptions
				Patients often reported somatic symptoms at first, but revealed depressive symptoms when probed.
				Causal beliefs
				Patients with depression (80%) attributed problems to psychosocial causes, compared with nearly as high a rate for those with somatoform diagnoses who attributed problems to somatic or physical causes (73.3%).
Qualitative interviews				
		Characteristics		
		Mean age 34 years		
		67.5% Women		
		71.2% Urban		
		82.5% Primary school		
		25% Secondary school		
		43.7% Housewives		
		78.7% Hindus		
Mirza et al. (2006)	To describe presenting symptoms and explanations from patient perspectives of GHQ positive cases attending a PC facility in Lahore	A semi-urban primary care centre in Pakistan	15 Consecutive patients of whom 11 were interviewed with the Short Explanatory Model Interview in Urdu after screening positive on the General Health Questionnaire	Illness descriptions
				Only 2/11 described their problems in psychological terms whereas 10/11 described physical complaints.
		Characteristics		Perceived impact
				10/11 described it as “very intense” and 9/11 said their mood was affected.
				Causal beliefs
				All respondents believed that psychosocial stressors caused depression.
		Mean age 34 years		
		10 Women		
		10 Married		

Qualitative interviews				
Shankar et al. (2006)	To delineate explanatory models of common mental disorders and their treatment as understood by traditional healers and patients	Christian Medical College Hospital in Vellore, India as well as interviews with healers in their homes	9 Traditional healers	58.3% had no diagnosis.
			7 Faith healers	Illness descriptions
		Characteristics		
		Mean age 49.9 years		
		61.1% Women		
		61.1% Married		
		41.9% Illiterate		
		65.6% Unemployed		
		90.3% Hindus		
			72 Patients identified consecutively from the healers	
				69% presented with psychological symptoms and 31% presented with physical symptoms.
				Perceived impact
				51% perceived problems as mild-moderate in seriousness and 30.6% believed that no treatment would help.
Qualitative interviews				Causal beliefs
				36% believed that their problems were caused by karma
Pereira et al. (2007)	To describe the explanatory models of illness in depressed women, their idioms of distress, and views of social circumstances	The catchment area of Aldona in Goa, India	35 Ever-married women with depressive disorder of whom 28 completed interviews	Illness descriptions
				Commonly reported symptoms were aches and pains, autonomic symptoms, palpitations, problems with sleep and appetite, reproductive problems.
		Characteristics		Causal beliefs
				Most common causes were attributed to economic factors, worries about the family, and gynaecological problems.
		Age range: 18–45 years		Perceived impact
				Functioning impacted through impaired relationships, problems with daily activities, and impaired sexual relationships.
Qualitative interviews				Self-help coping
				15 participants performed religious practices such as making offerings to God in the temple (Hindu), participating in a church mass (Christian), saying prayers or saying mantra in a temple/church or at home, and reading holy and spiritual books.
				6 Women shared problems with neighbours and family members.
Prameela et al. (2007)	To study the effectiveness of interventions in reducing depression	Self-Help Groups in North Karnataka, India	290 Women actively involved in microcredit and developmental programs out of whom 160 met cut-off scores on the General Health Questionnaire	Initially, many women physical symptoms to depression, but by sessions 5–7 many started to share emotional concerns.
				Illness descriptions
				Women preferred to describe their problems through physical aches and pains.
				Perceived impact
				Women expressed difficulties with managing household roles and providing suitable care to children.
		Characteristics		Causal beliefs
				Psychosocial stressors caused depression such as a husband׳s alcohol consumption, domestic violence, problems with in-laws, and loneliness.
		Mean age 34 years		Self-help coping
				Prayers and breathing exercises were the most frequently reported strategies.
		79.69% Married		
		80% Illiterate		
		94.82% from nuclear families		
Case-control study		Average 3 children		
		58.97% Low living standard		
Naeem et al. (2004)	To understand perceived vulnerability and restitution factors for depression	A lower middle class semi-urban community in Karachi	7 Married women with spontaneous recovery from depression	Illness description
				Patients preferred terms like “tension” or “pressure” to describe their problem.
				Causal beliefs
				The group regarded unemployment and poverty as main causes of depression.
				Self-help coping
				Sharing problems with family members or friends and praying were used as coping strategies.
		Characteristics:		
		Age range 22–40 years		
		All married		
		All had 1–8 children		
Qualitative interviews				
Savarimuthu et al. (2010)	To examine psychosocial causal factors of post-partum depression	12 Rural villages in Vellore, India	137 Post-partum women of whom 36 had depression.	26.3% were diagnosed with post-partum depression which was correlated with.
				Illness descriptions
				Women described depression in somatic terms.
				Causal beliefs
				A desire for abortion, unhappy marriage, physical abuse during pregnancy, a husband׳s alcoholism, and a desire for a boy but delivery of a girl were named as causes.
		Characteristics		
		97% Married		
		90.5% Illiterate		
		74.4% Housewives		
Qualitative interviews				
Chetna (2005)	To explore the beliefs of patients with depression	Academic department of the NIMHANS in India	Study group: 30 persons with depression	Illness descriptions
				The study group described depression as a problem with not being able to meet standards of perfection.
		Characteristics	Comparison group: 30 persons who were matched by age and sex.	
		Age range 18–50 years		
		Minimum education 7th standard		
Case-control study				
Sharma (1998)	To examine coping styles of patients with depression	Academic department of the NIMHANS in India	Study groups: 18 persons currently experiencing depression	Illness descriptions
				Patients with depression used terms to blame themselves for their circumstances.
				Causal beliefs
				The study groups believed that interpersonal problems with the family caused depression.
				Self-help coping
				Persons with depression frequently sought interpersonal support from family and friends and engaged in active problem solving.
		Characteristics		
			19 Persons recovered from depression	
			Comparison group: 40 normal subjects	
		Mean age 32 years		
		All had at least 10 years of formal education		
Case-control study				
Verma (1989)	To examine coping strategies and forms of social support in people with depression	Academic department of the NIMHANS in India	75 Women who scored more than 11 on the General Health Questionnaire	Illness descriptions
				Persons with depression often used terms that blamed themselves for their circumstances.
				Self-help coping
				Praying, family and friends, resignation at current circumstances, and engaging in problem solving were the most frequent methods of coping.
		Characteristics		
		Age range 18–45 years		
		All from rural areas		
Qualitative interviews				
Chandrashekhar et al. (2007)	To determine the prevalence of psychological morbidity, sources and severity of stress and coping strategies among medical students	Manipal College of Medical Sciences, Pokhara, Nepal	Medical students (*n*=525 with a response rate of 407 students or 75.8%)	The prevalence of psychological morbidity was 20.9%, higher among students of basic sciences, Indian nationality and whose parents were medical doctors.
				Illness descriptions
				Persons with depression often used terms that blamed themselves for their circumstances.
				Self-help coping
				Strategies most commonly used were positive reframing, planning, acceptance, active coping, self-distraction, and emotional support.
		Characteristics		
		Mean age 20.7 years		
		47.4% Women		
		49.1% Indians		
		40.7% Nepalese		
		10.2% Sri Lankan		
Survey				
Kermode et al. (2007)	To describe concepts about mental health and beliefs among women involved with a rural mental health programme	A community sample of women working with the Comprehensive Rural Health Project in Maharashtra, India	32 Women	Determinants of mental illness included interpersonal familial problems, having daughters and not sons, too many children or infertility, no freedom to move around, no independent income, violence, poor crops and drought.
				Illness descriptions
				Depression was frequently terms as “tension” or “pressure.”
Qualitative interviews				
		Characteristics		
		Mean age 44 years		
		Mean length of involvement in the programme was 18 years		

Kermode et al. (2009a, 2009b)	To understand local views of mental health and illness to inform a primary care based intervention	A low-resource setting in rural Maharashtra, India. Respondents were presented with a vignette on depression.	240 Randomly selected community adults based on cluster sampling	Illness descriptions
				Over 50% identified the condition as depression.
				Self-help coping
				Most respondents believed that social supports would help the depressed and named tonics, vitamins, and exercise as more helpful than visits to psychiatrists or medications.
Qualitative interviews				
			60 Purposively sampled village health workers	
		Characteristics		
		86% married		
		52% never attended school		
Kermode et al. (2009a, 2009b)	To understand local contexts in order to develop programs to change stigma and discrimination	A low-resource setting in rural Maharashtra, India	240 Randomly selected community adults based on cluster sampling	Illness descriptions
				Respondents believed that depression represented a sign of moral weakness that did not require medical interventions.
		Demographics		
			60 Purposively sampled village health workers	
		129 Women		
		86% Married		
		52% Attend school		
Qualitative interviews				
Suhail (2005)	To assess public mental health beliefs in Pakistan	3 Cities in Pakistan Punjab and their suburbs	1750 People semi-randomly selected (locales were not randomized, but every 10th household was approached); 901 responded	Causal beliefs
				Likely causes of depression as identified by respondents: virus (26.64%), allergy (21.98%), day-to-day problems (91.79%), adverse life events (71.25%), childhood problems (65.04%), genetic (44.17%), magic (14.32%), and moral weakness (62.49%).
		Demographics		
		Mean age 34 years		
		92.2% urban		
Survey				
Job (2004)	To examine coping strategies in patients with mental disorders	Academic department of the NIMHANS in India	Study group	Self-help coping
				People with depression frequently engaged in leisure activities and religious practices such as praying.
			40 Persons with depression	
			Comparison group	
			40 Normal subjects	
		Characteristics		
Case-control study				
		[Not available]		
Kaikhavani and Kumar (2005)	To examine coping strategies in patients with mental disorders	An outpatient clinic in Mysore, India	Study group	Self-help coping
			50 Persons with depression	Leisure activities, support from family and friends, cognitive restructuring, and problem solving were the most frequently reported coping strategies.
		Characteristics	Comparison group	
		Age range 25–65 years	50 Normal subjects	
Qualitative interviews				
Satija et al. (1998)	To examine the influence of stressful life events and coping strategies among persons with depression	An outpatient clinic in Mysore, India	Study group	Self-help coping
				Patients with depression frequently used avoidance as a coping strategy compared to normal controls.
			50 Persons with depression	
		Characteristics	Comparison group	
		Age range 15–60 years	50 Normal subjects	
		Over 45% 15–30 years		
		Over 18% women		
		30% Completed middle school		
Qualitative interview		58–70% Employed		
		Over 60% married		
		90% Urban		
		Over 90% Hindu		

**Table 2:** Characteristics of interviewed people with depression and their caregivers.

**Socio-demographic variables**		**Persons with depression (*****N*****=27)**	**Family caregivers (*****N*****=10)**
Age (in years)	Mean age (in years)	45	43
	Age range		
	17–29	3	1
	30–39	6	3
	40–49	8	3
	50 and above	10	3
Gender	Female	17	7
	Male	10	3
Education (highest completed)	No formal education	3	1
	Middle school or lower	9	2
	High school	9	2
	University	6	5
Occupation	In employment	11	4
	Not in employment	16	6
Clinical status	Recovered	11	Not applicable
	Not recovered	16	

**Table 3:** Culturally adapting psychological treatments (PTs) on the basis of consumer illness experiences.

	**Findings regarding consumer illness experiences**			
	**Illness descriptions**	**Causal beliefs**	**Illness impact**	**Self-help strategies**
Aspects of PT that the findings inform	Phenomenology	Negative life events and difficulties	Interpersonal problems	Distracting activities
	• Physical health complaints • Feelings of sadness, irritability, anxiety, hopelessness • Ruminative thinking • Low self-confidence • Disturbed sleep, concentration, appetite	• Interpersonal conflicts • Financial difficulties • Work and household stressors	Social and occupational impairments	Religious/spiritual practices
			Stigma and discrimination	Support from family and friends
			Other (health problems, finances, etc.)	Positive thoughts and acceptance of life׳s adversities
				Solving problems
				Adopting healthier lifestyles
				Relaxation
				Self-education
		Religious or spiritual beliefs		
		Physical or medical causes		
		Other causes (“moral weakness”, loneliness, failed aspirations, etc.)		

	Labelling			
	• Somatic terms • “Stress” and “tension” • Diagnostic, for example “depression” uncommonly			
What PTs can be adapted to South Asia?	Consider PTs such as interpersonal psychotherapy, behavioural activation and Cognitive Behavioural Therapy (CBT).	Consider PTs such as interpersonal psychotherapy and CBT.	Consider PTs such as interpersonal psychotherapy and CBT.	Consider PTs such as behavioural activation, interpersonal psychotherapy and CBT.
What should the goals of these PTs be?	PTs should target multiple symptoms, including somatic complaints, stress, disturbances in mood and functioning, and negative and ruminative thinking.	PTs should focus especially on identifying and modifying dysfunctional family and interpersonal relationships triggering depression, and addressing beliefs and myths regarding causality while respecting one׳s religious and cultural beliefs.	PTs should address multiple outcomes and build skills in patients to cope with various impacts of the illness.	PTs should encourage the use of self-help strategies that are beneficial to patients.
What should be the strategies emphasised within the PTs?	PTs can include behavioural activation, cognitive restructuring, psycho-education, problem-solving and relaxation training.	PTs can include addressing interpersonal triggers, supportive counselling, psycho-education to the patient and family, relaxation training and problem-solving.	PTs can include addressing interpersonal triggers, behavioural activation, enhancing social networks, psycho-education to the patient and family, relaxation training, problem-solving cognitive restructuring and referrals to health services.	PTs can include behavioural activation, psycho-education to the patient and family, enhancing social networks, cognitive restructuring, relaxation training and problem solving.
What should the content of the PT be?	PTs should use terminology and explanations for the illness that are consistent with patients׳ descriptions of the illness (for example, “tension”, “stress”). Psychiatric terms like “depression” can be replaced with these more culturally appropriate terms.	Psycho-education needs to explain the link between stress and depression, and how coping with stressful situations (especially interpersonal) may improve symptoms.	PTs should provide information on myths and misconceptions about the illness.	Therapists should encourage patients to follow forms of coping that helps them (e.g., adopting healthier lifestyle).
			PTs can involve family and significant others by educating them about the impact of depression and what they can do to help reduce its effect on interpersonal relationships and how they can replace negative/ stigmatising behaviours with more positive ones.	
				PT strategies, for example, behavioural activation, should be modified so that their content and form are in line with forms of coping that are culture specific (e.g., religious/spiritual coping).
		It can use culturally appropriate materials and illustrations to depict stressful situations relevant to the local community, for example diagrams with characters depicting family (for example, husband-wife) conflict.		
				These coping strategies can be methods/techniques within a PT strategy; for example, prayer as a form of behavioural activation, or yoga/ breathing exercises as techniques for relaxation training
			PTs, for example through problem-solving, should focus on imparting skills to patients to handle interpersonal and other problems effectively.	
				Vignettes of coping strategies from commonly used religious texts can be used to explain strategies and encourage following them. Inspirational quotes from self-help books by local authors / revered members of the local community can also be used for the same purpose.
			Patients should be encouraged to ask family and friends for help and support.	
				PTs can enhance social support for patients, for example by using existing support systems (family) or referring to local self-help/ social networks in the community (example, religious groups).
		PTs can educate family and significant others about how interpersonal and other stressors can affect depression and involve them in reducing these forms of stress.		
	Psycho-education needs to explain the link between the mind and the body using examples of physical complaints most commonly reported (i.e., headaches, fatigue, reproductive complaints).			
		PTs, for example through psycho-education and problem-solving, should also help build skills to handle interpersonal and other conflicts or stressors effectively.		
		Psycho-education should contain information on myths and misconceptions regarding the illness.		
	Psycho-education and other strategies need to provide patients and families with information on the illness, and ways of coping with it, for example, ways of reducing stress or dealing with physical health complaints			
	Manuals and treatment materials need to be translated into local languages, having explanations and descriptions of the illness that capture local idioms.			
What characteristics should the person who delivers the PT have?	The person should be fluent in the local language and familiar with expressions of the illness in the community.	He/she should be familiar with commonly held beliefs about the illness in the community.	He/she should be a member of the local community, likely to experience many of the social problems of patients, and serve as role models for reducing stigma	He/she should be familiar with local customs and traditions (for example, family roles) and ways of coping (for example, religious practices).
